# A Review of the Application Research on Inorganic Clay Minerals Synergising with Bio-Based Flame-Retardant Systems to Enhance Polymer Performance

**DOI:** 10.3390/polym18121487

**Published:** 2026-06-13

**Authors:** Shihao Zheng, Yong Liu, Fang Zhou, Hao Yuan

**Affiliations:** 1 School of Resource & Environment and Safety Engineering, Hunan University of Science and Technology, Xiangtan 411201, China; 2 Engineering Research Center for Fire and Explosion Prevention Materials and Equipment in Underground Spaces, Xiangtan 411201, China; 3 Key Laboratory of Fire and Explosion Prevention and Emergency Technology in Hunan Province, Xiangtan 411201, China

**Keywords:** inorganic clay minerals, bio-based flame-retardants, synergistic flame-retardant system, polymer, flame-retardant mechanism

## Abstract

In recent years, synergistic effects between inorganic clay minerals (e.g., montmorillonite, sepiolite, kaolinite) and bio-based flame retardants (e.g., chitosan-based, lignin-based, phytate-based) have achieved certain progress in the area of polymer flame retardancy. The effects of bio-based flame retardants are exerted through mechanisms such as catalytic char generation and vapour-phase hindrance. However, they have limitations when used alone, including insufficient thermal stability and the need for a high dosage. Inorganic clays form physical barriers through their layered or tubular structures. The high thermal stability of these structures suppresses heat and mass transfer, thereby offsetting the shortcomings of bio-based flame retardants. This synergistic combination greatly improves the flame retardancy of polymer composites, often strengthening their mechanical performance in the process. It therefore offers great potential for the design of multifunctional, eco-friendly flame-retardant polymer composites. Nevertheless, a systematic review of the synergistic mechanisms, fabrication approaches and application progress of different inorganic clay minerals when combined with various bio-based flame retardants is still lacking. Therefore, this article offers a comprehensive review of the current developments of synergistic systems that incorporate various primary clays, such as sepiolite and montmorillonite, with bio-based flame retardants for usage in polymers. Before this, the synergistic flame-retardant mechanism and the key preparation techniques of the composite system were explained in detail. Finally, this article puts forward solutions to the current challenges and sets out prospects for innovation in the designing of flame-retardant materials and the optimisation of processes. The aim is to promote the sustainable growth of efficient, eco-friendly flame-retardant materials.

## 1. Introduction

Polymer materials are extensively applied in the construction, electronics and transportation sectors thanks to their remarkable properties, such as being lightweight and easy to process. However, their structure contains significant quantities of combustible elements, including carbon, hydrogen and oxygen. This renders traditional polymer matrices unsuitable for use as refractory materials, particularly in applications involving high-temperature environments. Moreover, most polymers exist as linear or weakly cross-linked chains rather than stable three-dimensional gel-like networks, making them inherently flammable. Their flammable nature further restricts their use in sectors that require high flame-retardant properties, such as aerospace, electronics, semiconductors and transportation [1,2]. In the event of a fire, polymers decompose readily at high temperatures, burn easily and release toxic fumes and harmful gases accompanied by melting and dripping. This poses a substantial risk to human health and property while also having a detrimental impact on the environment [3]. Therefore, boosting the flame retardancy and smoke suppression of polymer composites, especially the construction of thermally stable cross-linked polymer gel networks, is of significant importance to improve fire safety and reduce hazards to human health and the ecosystem.

The advancement of flame retardancy in polymer composites to meet the demands of high-performance applications is the driving force behind advancements in flame retardant technology. This represents a significant challenge for the industry in the field of flame retardancy. Many traditional flame retardants developed by researchers pose hazards to the environment and human health, such as polybrominated diphenyl ethers (PBDEs) [4,5,6], hexabromocyclododecane (HBCD) [7], tetrabromobisphenol A (TBBPA) [7] and other brominated flame retardants. Short-chain chlorinated paraffins (SCCPs) [8,9] and dechlorane plus [10,11] are representative chlorinated flame retardants. The US National Institute of Environmental Health Sciences (NIEHS) states that halogenated flame retardants, such as HBCD, can enter the food chain through leaching [12]. This can cause endocrine disruption and neurotoxicity in humans. Research projects published by the United States Environmental Protection Agency (EPA) indicate that HBCD (a brominated flame retardant) exhibits long-term and long-distance migration properties in the environment [13]. Based on the results of tests conducted on animals, HBCD demonstrates high toxicity to aquatic organisms and has the potential to impact human reproduction, development and the nervous system [14]. Traditional inorganic flame retardants, such as magnesium hydroxide (MH) [15] and aluminium hydroxide (ATH) [16], are regarded as environmentally friendly additives. However, on account of its minimal flame-retardant effectiveness, high loading levels of 30–65 wt% are typically required to meet flame-retardant requirements. This results in the polymer material having diminished mechanical properties and processing performance [17,18].

In the current global context, non-renewable resources such as oil, natural gas and coal are becoming increasingly scarce. The prevailing future trend is to utilise renewable resources, such as biomass energy, solar power and wind energy, to meet energy consumption demands. Against this backdrop, the active exploration of highly efficient, environmentally friendly and sustainable bio-based flame retardants has become a significant challenge in materials science. It is essential to research their impact on the flame retardancy, thermal stability and smoke suppression properties of polymeric materials without compromising the mechanical properties of the polymer matrix in order to develop effective, eco-conscious flame-retardant systems.

In recent years, the synergistic application of inorganic nanoclay minerals (e.g., montmorillonite [19], kaolinite [20], and sepiolite [21,22]) with bio-based flame retardants (e.g., chitosan [23], phytate [24], and lignin [25,26]) has demonstrated unique advantages. For instance, research indicates that the montmorillonite–phytate composite system in polybutylene succinate (PBS) reduces the heat release rate (HRR) by over 50%, achieves a limiting oxygen index (LOI) of up to 31.4% and greatly increases the residual carbon content. Concurrently, incorporating modified montmorillonite enhances the flexural and impact strengths of the PBS composite material [27]. Montmorillonite acts as a synergistic flame retardant by forming a dispersed nanoscale structure within polymers through intercalation or exfoliation. This promotes the formation of a dense char layer during combustion that blocks the transfer of heat, combustible material and oxygen [28]. Furthermore, flame retardants derived from phytate enhance flame retardancy through mechanisms such as acid-catalysed dehydration, which forms carbon, and the release of substances that scavenge free radicals [29,30].

Table 1 summarises the flame-retardant performance of different composite materials, presented through key flame-retardant test parameters, to highlight the synergistic flame-retardant properties of inorganic clay minerals and bio-based flame retardants in various polymers, including polylactic acid (PLA), PBS, polyurethane acrylate (PUA), acrylic fibre, epoxy resin (EP), cotton fabric and polypropylene (PP). The key parameters reported are the limiting oxygen index (LOI), UL-94 rating, reduction in peak heat release rate (pHRR), reduction in total heat release (THR), and total smoke production (TSP). The LOI values range from 27 to 36.5, and several systems (e.g., 10DOPO-SEP/PLA, 22I/3LM/PBS, 20LDHs@PA-MEL/PP and LDH-LS/PP) achieve a V-0 rating. The pHRR reduction varies between 34.1% and 74.6%, with the highest value observed for PI-1.0MK in PUA (74.6%). The THR reduction falls in the range of 19.6–49%, and the lowest TSP value (33.8 m^2^) is recorded for the 22I/3LM/PBS system. Overall, these data demonstrate that the combination of inorganic clay minerals with bio-based flame retardants effectively reduces heat release and smoke production across a broad range of polymers, highlighting their synergistic flame-retardant efficiency. Table 2 summarises the synthesis routes and brief explanations of the synthesis schemes for inorganic clay mineral/bio-based flame-retardant systems.

**Figure 1:**
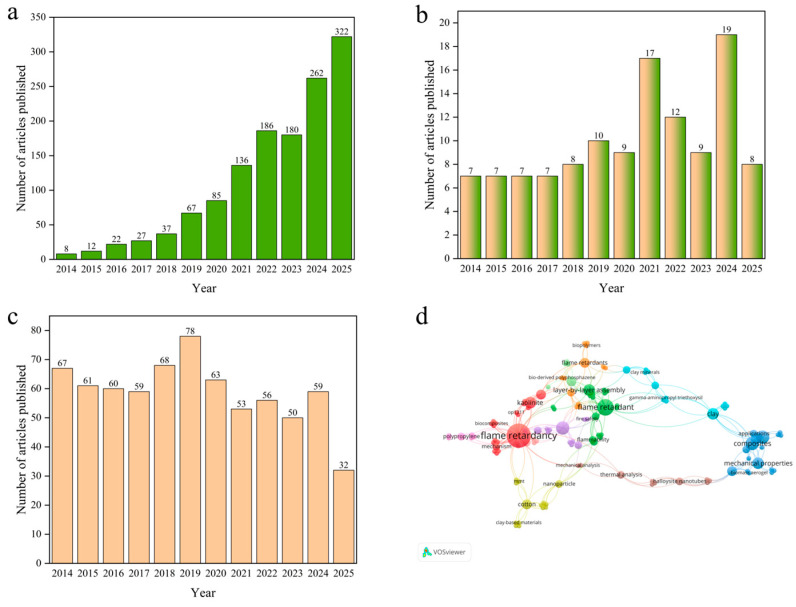
Statistics on the number of papers published on flame retardants including (**a**) bio-based, (**b**) bio-based/clay, and (**c**) clay. Bio-based clay flame retardant’s visualisation of (**d**) co-occurrence keyword.

**Figure 2:**
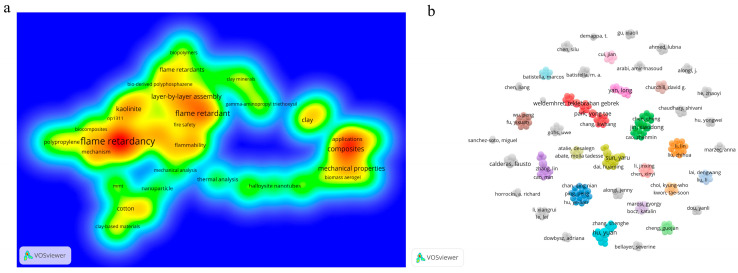
(**a**) Density visualisation of keywords, and (**b**) co-authorship network.

However, the utilisation of synergistic flame-retardant systems that combine inorganic nanoclay and bio-based flame retardants in polymer composites is still in its infancy. Nevertheless, as the number of polymers flame-retarded by bio-based or clay flame retardants increases, so has the overall application of their composite systems. As Figure 1a–c illustrate, the most rapidly expanding sector is bio-based flame retardants, while research activity related to bio-based clay flame retardants increased in 2024, although these products have also demonstrated an upward trend in recent years. Keyword analysis in Figure 1d and Figure 2a indicates that bio-based clay flame-retardant systems also hold significant research value. This is further evidenced by existing research in this area by numerous authors, as depicted in Figure 2b. Many contributors have made substantial contributions to this body of work. However, based on the research findings currently published, optimising synergistic flame-retardant systems still presents multiple challenges, such as dispersion difficulties [40,41]: unmodified nanoclay particles have a large particle size and a high impurity content. They also have elevated surface energy and hydrophobicity, which results in poor compatibility at the polymer interface. Consequently, they are difficult to disperse uniformly within the polymer matrix and tend to agglomerate. They have insufficient thermal stability: bio-based flame-retardant agents tend to decompose prematurely at high temperatures, diminishing their effectiveness as flame retardants. However, their thermal stability can be improved by combining them with nanofillers. The flame-retardant mechanism is complex: the mechanisms involving physical barrier effects and chemical catalysis associated with inorganic nanoclay and bio-based flame retardants are unclear. Potential mutual interactions require further in-depth investigation [42].

Recent reviews have examined the application of various mineral fillers in flame-retardant polymers—for example, work on fire-resistant polymer composites based on mineral fillers [43] and a review on clay-reinforced PVC composites [44]. However, these studies largely focus on single-component mineral systems. As a result, a systematic account of the synergistic combinations of clay minerals with bio-based flame retardants remains missing, and the underlying mechanisms of synergy have not been explored in depth. The present work aims to fill this gap. This systematic review examines the synergistic flame-retardant mechanisms of various inorganic clay and bio-based flame retardants. It analyses the flame retardancy and smoke suppression properties of their constituent binary flame-retardant systems when applied to different polymeric matrices (thermoplastics and thermosets). The review provides scholars with a clearer understanding of how binary or multi-component flame-retardant systems can enhance polymer properties synergistically, compensating for the limitations of single flame retardants. This review aims to meet the demands for developing high-performance, cost-effective, and multifunctional flame-retardant composite materials in future emerging industries, advance the industrialisation of key green flame-retardant technologies, and provide a theoretical foundation for further research.

## 2. The Synergistic Flame-Retardant Mechanism of Inorganic Clay Minerals and Bio-Based Systems

### 2.1. Inorganic Clay Mineral

When employed as flame-retardant additives in polymer nanocomposites, inorganic clay minerals exhibit a fundamentally distinct flame-retardant mechanism compared to conventional flame retardants. They primarily function as a physical barrier, inhibiting the exchange of combustible substances between the polymer matrix and its surroundings. They also block heat transfer and impede oxygen circulation, thereby achieving flame retardancy [45]. This is the primary flame-retardant mechanism of mineral clay. Furthermore, it reduces the flowability and drip resistance of polymer melts, which prevents the spread of burning material [46]. Conversely, the radical trapping capacity is very limited. For example, when iron-containing compounds in clay minerals undergo thermal decomposition, iron atoms or low-valent iron oxides are released into the gas phase, creating an efficient catalytic cycle. This mechanism acts like a “molecular sponge”, rapidly absorbing the hydrogen–oxygen radicals that sustain the flame and thereby inhibiting chain combustion reactions [47]. However, this typical vapour-phase flame retardancy mechanism is only effective at sufficiently high concentrations of iron compounds, making it a rather negligible form of vapour-phase flame retardancy. Table 3 summarises the primary mechanisms of inorganic clay minerals.

**Figure 3:**
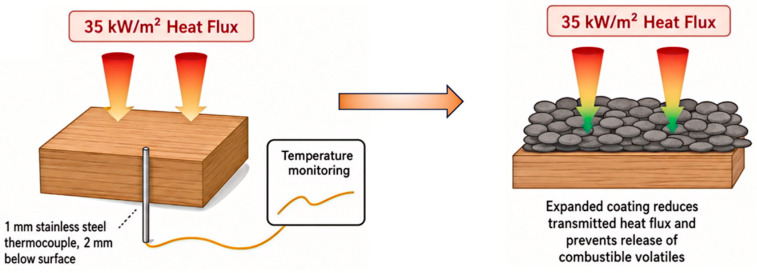
Schematic diagram of simple combustion changes of coated wood [49] (redesigned by authors).

In recent years, researchers have focused on using inorganic clay minerals to enhance polymer properties. Taking montmorillonite as an example, Carosio et al. [49] produced stand-alone clay nanopapers made from montmorillonite nanosheets and water-soluble polymers. These were then used as fire-retardant coatings on wooden surfaces. The highly ordered clay-packing structure of clay nanopaper enables it to isolate oxygen and heat while preventing the escape of degradation products. As shown in Figure 3, temperature measurements taken inside a cone calorimeter demonstrate that the expanded structure formed by the coated timber sample reduces the heat flux from the calorimeter heater. Consequently, the temperature rises more slowly than it would with uncoated timber. When suitably modified, organic montmorillonite (OMMT) can also act as a synergist to increase the flame retardancy of polymers in conjunction with IFR (composed of APP and MA in a 2:1 mass ratio). This involves the OMMT layers migrating and accumulating on the surface of the carbon particles. There, they function as physical or chemical cross-linking agents, reinforcing the carbonaceous layer and stabilising the char residue. It can form an expanded carbon layer within the condensed phase that is reinforced, exhibiting enhanced strength, a more compact structure and superior thermal insulation properties. This achieves comprehensive performance optimisation of polymer materials. This research has been corroborated by Wang et al. [50].

### 2.2. Bio-Based Flame Retardant

Bio-based flame retardants operate primarily in the condensed phase through multiple synergistic mechanisms. Their key strength is in achieving fire resistance in an eco-friendly way by creating high-quality char layers for fire barriers, along with methods like vapour-phase dilution, free radical capture, and endothermic cooling [51,52,53]. However, the use of bio-based flame retardants is limited by factors such as low thermal stability and a tendency to decompose prematurely, which compromises their effectiveness as flame retardants. Current efforts are focused on addressing this issue by using molecular design and compounding techniques to improve the efficiency of flame retardancy. Similarly, in line with the primary flame-retardant mechanism of inorganic clay minerals, the char layer formed effectively insulates the substrate from heat and prevents the diffusion of oxygen and combustible degradation products. This interrupts the combustion heat cycle. As many bio-based flame retardants (e.g., phytate, starch, phosphate ester derivatives) are inherently rich in phosphorus, they can generate phosphates and polyphosphates during pyrolysis. These acidic substances can catalyse the dehydration, cross-linking and aromatisation of oxygen-containing polymers or carbon sources. This promotes the formation of dense coal beds with a high carbon content, which can be either expansive or non-expansive [54,55]. Moreover, bio-based materials themselves can serve as carbon sources, as they are rich in carbon, hydrogen, and oxygen and are polyhydroxy compounds such as lignin, cellulose, starch, and chitosan. They undergo esterification and dehydration reactions under the catalysis of acid sources, thereby forming a stable carbon skeleton [56,57].

Bio-based flame retardants release gaseous products during pyrolysis that can disrupt combustion chain reactions, forming a gas-phase flame retardation mechanism. Upon thermal degradation, they release large amounts of non-combustible gases (e.g., H_2_O, CO_2_ and NH_3_), which effectively reduce the concentration of combustible gases within the flame combustion zone while absorbing heat and cooling the combustion temperature [58,59]. Although most bio-based molecules have weaker capabilities than highly efficient halogenated flame retardants for capturing highly reactive H· and HO· radicals, certain nitrogen-containing bio-based materials—such as the heterocyclic pyrolysis products of chitosan—are suspected to release nitrogen-containing radicals in the gas phase. This exerts a certain inhibitory effect on combustion chain reactions [60,61]. Table 4 provides an overview of several common bio-based flame retardants and their main mechanisms.

Nylon–cotton blended fabrics have a broad spectrum of applications, covering military uniforms and civilian textiles. Nevertheless, cotton is a flammable cellulose fibre. The process of enhancing the flame-retardant properties of nylon is complicated by the thermoplastic nature of this material, which melts and drips during processing. To address this challenge, Kulkarni et al. [74] utilised bio-based materials, TA and PA, to impart flame-retardant properties to nylon–cotton blended fabrics. Research has found that, under thermal action, PA decomposes to generate phosphoric acid, which strongly catalyses the dehydration cross-linking of TA and cellulose to form a phosphorus-rich carbonaceous skeleton. Concurrently, TA’s pyrolysis products generate a high carbon content, while also quenching free radicals in the gas phase. The intermolecular forces between PA and TA ensure they interact uniformly in the condensed phase. Together, they form an expanded carbon layer that offers excellent thermal insulation, an effective oxygen barrier and smoke suppression properties.

**Figure 4:**
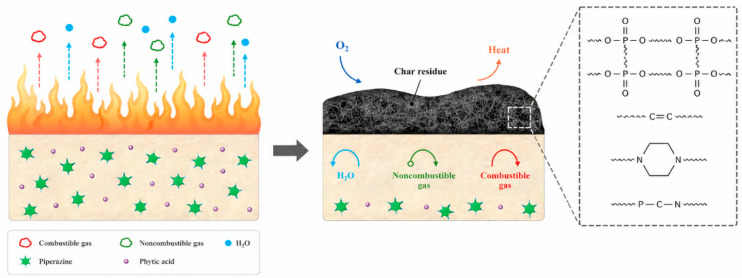
The flame-retardant mechanism of PHYPI in PP [75] (redesigned by authors).

Gao et al. [75] synthesised a novel bio-based phytate salt (PHYPI) via a salt reaction involving phytic acid and piperazine. In the process of producing flame-retardant PP, it exhibited high flame-retardant efficiency by relying on the stable carbon layer of the condensed-phase and gas-phase dilution cooling. During initial heating, phytic acid in PHYPI undergoes rapid dehydration, resulting in the rupture of ionic bonds. At the same time, non-combustible gases such as H_2_O and CO_2_ are released and thermally stable cross-linked structures, including P-N-C and P-O-C, are formed. Furthermore, phytic acid decomposes further at elevated temperatures, forming acidic compounds such as pyrophosphate. These compounds strongly promote the dehydration cross-linking of polypropylene, forming a dense carbon layer. Figure 4 presents the conclusions regarding the relevant flame-retardant mechanisms.

### 2.3. Inorganic Clay Minerals/Bio-Based Synergistic System

The combination of inorganic clay minerals and bio-based flame retardants overcomes the limitations of conventional single flame retardants through a dual flame-retardant mechanism involving both the condensed phase and gas phase. This approach enhances the flame-retardant efficiency of polymer materials while maintaining mechanical properties, thereby addressing the hardness deficiency associated with traditional flame retardants in engineering plastics. During polymer combustion, the layered and chain-like structures of inorganic clay minerals encourage the formation of dense char. This isolates the circulation of combustible material within the polymer matrix, both internally and externally. This establishes a robust physical barrier by preventing the free movement of oxygen, combustible substances, and reactive free radicals [76,77,78]. Their crystal structures predominantly consist of alternating silicon–oxygen tetrahedra and metal–oxygen octahedra. This unique crystalline arrangement also gives the polymeric materials outstanding thermal stability [45,79]. Bio-based flame retardants operate via a synergistic mechanism involving condensed-phase and gas-phase radical quenching. Upon thermal decomposition, they generate substances that capture active radicals and produce inert materials that can dilute combustible gases. These include phosphorus-containing radicals (P·, PO· and ·PO_2_), as well as inert gases such as CO_2_ and H_2_O. These substances substantially reduce the concentration of combustible gases, thereby significantly inhibiting chain combustion reactions [80], terminating the material’s exothermic combustion process.

Zhang et al. [81] successfully produced a rigid polyurethane foam (RPUF) by co-modifying it with the MMT and chicken feather protein (CF). This was achieved using the special layered structure of the MMT and the stable chemical bond formed by the one-step, all-water foaming method. Both experimental design and theoretical calculations reveal that the sample’s flame retardancy is enhanced by the collective action of the MMT and CF. This flame-retardant mechanism relies on the physicochemical properties of the MMT and the P-O-Si bonds formed with the protein in the chicken feathers. When the MMT is exposed to high temperatures, the water molecules adsorbed between its layers absorb heat and evaporate to form an insulating layer of water vapour. Simultaneously, the CF protein contains a high concentration of phosphorus and nitrogen, which react with the Si-OH groups on the MMT surface to form P-O-Si bonds [82], jointly promoting the formation of a carbon layer. Zhi et al. [83] synthesised a vanillin-based, flame-retardant epoxy resin (VH-HDA-EP), which features a rigid Schiff base structure and flexible alkane segments. The starting materials used were vanillin, 1,6-hexanediamine, and epichlorohydrin. The VH-HDA-EP/DDM thermosetting resin was obtained by curing with 4,4′-diaminodiphenylmethane (DDM). The TG analysis shows that the residual carbon content in an atmosphere of nitrogen is 30.8 wt%, whereas the residual carbon content of DGEBA/DDM is only 17.9 wt%. Compared to the latter, the VH-HDA-EP/DDM exhibits reductions in THR, TSP and maximum smoke density of 45.1%, 73.4% and 36.2%, respectively, while achieving a V-0 rating and increasing LOI to 38.5%. Analysis of the char residue shows that the VH-HDA-EP/DDM Schiff base structure forms a continuous, dense carbon layer during combustion, which improves the material’s flame-retardant properties.

**Figure 5:**
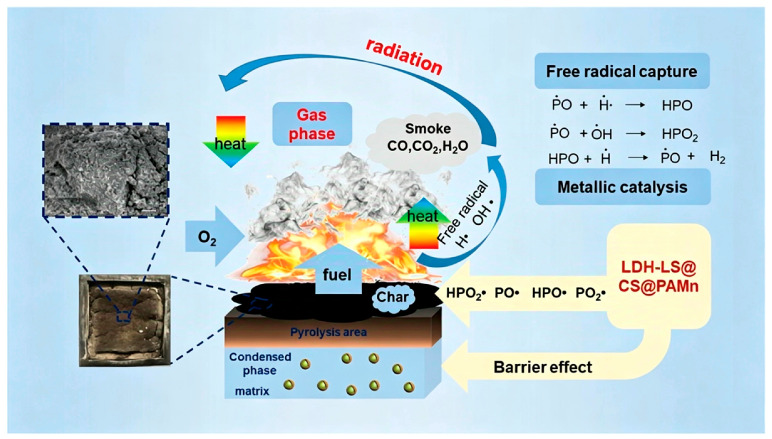
Yan et al. explore the potential mechanism of bio-based inorganic clay flame retardants [84] (provided by American Chemical Society and Copyright Clearance Center. License Number 6281790702669).

Yan et al. [84] used a layer-by-layer self-assembly approach with water as the medium to synthesise the bio-based flame retardant LDH-LS@CS@PAMn. This was then used to investigate novel methods of achieving green and synergistic flame retardancy in polypropylene. This flame-retardant system employs a synergistic design of LDH nanosheets (physical barrier)—bio-based components (catalysing char formation and cross-linking)—synergistic multi-element flame retardancy (P/N/S/Mn). Mn^2+^ catalyses the formation of additional P-O cross-linking structures, collaborating with the condensed phase during combustion to construct a robust char layer and employing vapour-phase flame retardancy to disrupt combustion reactions. This creates a synergistic physicochemical flame-retardant barrier that significantly enhances the flame-retardant and smoke suppression properties of PP composite materials. This can be seen intuitively from the mechanism diagram in Figure 5.

## 3. Preparation of Inorganic Clay Mineral/Bio-Based Flame-Retardant Composites

### 3.1. Solution Intercalation Method

The solution intercalation method is a technique used to prepare intercalated composite materials. It uses chemical or physical forces within a solution, typically water or an organic solvent, to insert external molecules, ions or polymer chains into the interlayer spaces of inorganic clay minerals such as montmorillonite or kaolinite [85,86,87]. Its primary function is to modify the interlayer spacing and interfacial properties of clay materials, enabling dispersion and functionalisation at the nanoscale. Several examples demonstrate that, when layered materials such as montmorillonite and LDH are added to a suitable solvent and dispersed thoroughly by stirring or ultrasonication, the solvent molecules first penetrate the interlayers. This causes the sheets to expand slightly. These conditions are then conducive to the subsequent insertion of target bio-based flame retardants (e.g., phytate and chitosan) as intercalation agents. Moreover, in solution environments, intercalants penetrate and displace lamellar layers via specific interactions. The solid product is then separated by centrifugation and filtration. After washing and drying, the interlayer molecules become immobilised and the spacing between layers increases.

The key operating principle of solution intercalation is that the intercalation process is primarily driven by ion exchange, hydrogen bonding, van der Waals forces, hydrophobic interactions and acid-base reactions/coordination bonds. Each of these forces has a different mechanism of action and typical application scenario. Of these, ion exchange is the most common driving force. It achieves intercalation by exchanging interlayer-exchangeable ions, such as Na^+^ in montmorillonite and CO_3_^2−^ in LDH, for organically bound, biologically charged ions in solution [88]. Hydrogen bonding relies on polar functional groups (e.g., -OH, -NH_2_, C=O), forming a network of hydrogen bonds with corresponding groups on the surface of the clay layer to achieve fixation [89]. Van der Waals forces and hydrophobic interactions arise from the aggregation of the hydrophobic segments of long-chain organic molecules within the hydrophobic interlayer regions of clay as these molecules seek to avoid polar solvents [90]. Acid-base reactions and coordination bonds are formed through electron transfer between intercalating agent molecules and coordination or acid-base interactions with metal ions (e.g., Al^3+^ and Mg^2+^) on the surface of the clay layer, resulting in intercalation [91].

### 3.2. Co-Precipitation Method

The co-precipitation method is a classic solution-based chemical synthesis technique. It involves achieving a state of supersaturation for two or more soluble components (typically metal cations) within a homogeneous solution, under the influence of a precipitating agent. The components then co-precipitate according to specific stoichiometric ratios, forming a solid precipitate with a uniform composition and a controllable structure (usually a precursor, a solid solution or a composite oxide) [92,93]. In inorganic clay/bio-based flame-retardant systems, the co-precipitation method is one of the most important ways of synthesising layered double hydroxides (LDH) and bio-based, molecularly intercalated LDH. A solution containing target divalent/trivalent metal cations (e.g., Mg^2+^ or Al^3+^) is mixed with a solution bearing bio-based anions (e.g., phytate). Under alkaline conditions, co-precipitation occurs, forming LDH with the bio-based molecules intercalated directly [94,95,96].

The one-step co-precipitation method of bio-based anion intercalated LDH is an important method for preparing inorganic clay/bio-based flame-retardant composite materials. The key process involves the simultaneous titration and mixing of solutions containing bio-based anions, such as PA and lignin sulfonate (LS), with solutions of Mg^2+^/Al^3+^ metal salts and a NaOH precipitant. Precise control of critical process parameters enables the efficient preparation of the target product [97,98,99,100].

This method has significant advantages. Firstly, bio-based anions are directly embedded into the interlayer during the LDH crystallisation process, achieving uniform molecular-level recombination and effectively solving the problem of incomplete molecular intercalation, maximising the interfacial bonding effect. Secondly, by regulating the ratio of metal ions and the structure and precipitation conditions of bio-based molecules, multifunctional integrated designs that encompass flame retardancy, catalysis and smoke suppression can be achieved. Finally, it facilitates the direct formation of highly thermally stable nanofillers in a single step, thus avoiding the excessive impurities produced by multi-step processes. In summary, the co-precipitation method provides a technically sound and efficient way of preparing intercalated LDH materials alongside other high-performance bio-based materials. It also establishes a fundamental paradigm for the controlled synthesis of green flame retardants, thus advancing the development of high-performance composite materials further.

### 3.3. Hydrothermal Method

The hydrothermal method, also known as the solvothermal method, is a process that takes place in a sealed pressure vessel, typically a high-pressure reactor, in which water or other solvents act as the reaction medium. Heating to elevated temperatures (usually above the normal-pressure boiling point of water, which is 100 °C) establishes a high-pressure subcritical or supercritical reaction environment. This facilitates the dissolution and recrystallisation of substances that are poorly soluble or insoluble under ambient conditions, or catalyses chemical reactions in precursors. Ultimately, this method synthesises nanomaterials characterised by high crystallinity, specific morphologies, and dimensions [101,102,103,104].

The hydrothermal method plays a significant role in the preparation of inorganic clay/bio-based flame-retardant composites. Two primary synthetic approaches are employed to achieve the controlled synthesis of high-performance bio-based intercalated LDH materials. The first step is to synthesise and crystallise high-quality LDH. This involves preparing amorphous or low-crystallinity precursor gels using co-precipitation or ion exchange methods. These are then subjugated to a hydrothermal treatment process within a high-pressure reactor. This procedure significantly improves the crystallinity of the LDH, optimising the orderly arrangement of the crystal layers and enhancing thermal stability and physical barrier properties. It simultaneously achieves precise control over morphology and dimensions while promoting the deep intercalation of large bio-based anions (e.g., phytate ions) and stabilising the layered structure [105,106]. The second approach uses a one-step, in situ synthesis strategy. This method involves mixing metal salt solutions and bio-based molecules (such as sodium phytate) with a precipitating agent in a reactor vessel to facilitate a hydrothermal reaction. Using a high-temperature, high-pressure environment simultaneously drives the crystallisation of LDH and the intercalation of bio-based molecules, producing a highly crystalline composite product in one step. Compared with the two-step method of co-precipitation followed by hydrothermal treatment, this method offers the advantage of a more direct and efficient preparation process [107,108].

### 3.4. Physical Adsorption Method

Physical adsorption is a preparation or separation process in which target substances (adsorbates) become reversibly attached to the surface or pores of solid materials via physical forces, primarily van der Waals forces but also including hydrogen bonds and electrostatic attraction. Its defining feature is that it does not involve any chemical reactions, such as electron transfer or the formation of new chemical bonds [109,110,111]. When preparing composite materials by combining inorganic clay minerals with bio-based flame retardants, the physical adsorption method is a simple approach. The main aim is to achieve effective composite formation between the bio-based flame retardant and the inorganic clay by applying physical forces. The typical operational procedure for this method may be as follows: First, disperse the montmorillonite and illite in water or other solvents. Next, introduce solutions of bio-based flame retardants, such as chitosan and phytate, into the system. The mixture is then stirred for a specified duration under the appropriate conditions, including the correct temperature and pH levels. This process enables the bio-based molecules to adsorb onto the outer surfaces, edges or pore entrances of the clay particles through physical interactions. Finally, the solid product is separated via centrifugation or filtration. After washing and drying, the target composite material is obtained [112,113].

Physical adsorption has clear advantages and notable limitations. Its strengths lie in its simple operational procedures, which require no stringent reaction conditions and have a low experimental threshold. Furthermore, it preserves the chemical structure and biological activity of bio-based molecules during the composite process, making it ideal for rapid formulation screening in a laboratory setting. However, this approach has significant limitations that cannot be overlooked. The main problem is that the bonding between bio-based molecules and inorganic clay only occurs through physical interactions. These forces are weak and unstable. Under the high temperatures (around 200 °C) and high shear forces encountered during polymer melt processing, delamination, migration or decomposition can easily occur. Consequently, the efficiency of flame retardancy is significantly diminished during processing and subsequent use. At the same time, bio-based molecules bound to inorganic clay minerals are confined to surface adsorption. This results in low loading capacities and uneven distribution, and also hinders the formation of stable, close-knit nanoscale interactions between these molecules and the clay layers. Consequently, the synergistic flame-retardant effect arising from catalytic carbonisation and physical barrier formation is not realised to its full potential.

### 3.5. Layer-by-Layer Self-Assembly Method

Layer-by-layer self-assembly (LBL) is a technique of constructing multilayer nanocomposite films. This process relies on reversible interactions at interfaces to sequentially deposit distinct functional components onto the surface of a solid substrate. This process involves the deposition of alternating layers, each comprising a single molecular or nanometre-thick layer. This allows for accurate adjustment of the film’s structure and properties [114,115,116]. The core principle of LBL is a highly standardised, cyclic, iterative immersion-washing process. This process operates on the basis of alternating component adsorption, which is driven by electrostatic interactions and charge reversal effects: First, the substrate material is pretreated to give it an initial negative charge. Next, the substrate is dipped in a solution containing component A, which carries an opposite charge, and left to stand. Component A then adsorbs onto the substrate surface via electrostatic attraction, thereby reversing the surface charge. Any excess physically adsorbed component A is then removed by rinsing with pure water. Next, the substrate is transferred into a solution containing component B, which has an opposite charge to component A. Component B then adheres to the surface of the A layer through electrostatic attraction, which reverses the substrate’s surface charge once more. Finally, the substrate is rinsed again with pure water. Repeat the aforementioned adsorption–pure water washing cycle steps. Each cycle adds one A/B bilayer to the substrate surface. After repeating this process iteratively to achieve the target number of layers (n), a regular layered composite structure with a (A/B)^n^ substrate is formed [117,118,119,120].

LBL is a surface engineering technique that involves programmed cyclic operations. This process precisely constructs multilayer nanocomposite films at solution interfaces through electrostatic interactions [121,122,123]. This method is highly valuable in the area of flame retardancy owing to its ability to integrate bio-based and inorganic components at the nanoscale to form core–shell composite materials with a defined structure. This ordered structure is an ideal model for explaining the sequential release of components and the synergistic pathways of carbonisation during combustion. It overcomes the limitations of simple physical blending systems. Furthermore, its combination of high efficiency and durability makes it particularly well-suited to the surface flame-retardant treatment of flexible products, such as textiles and foams. This opens the path to the practical implementation of green flame-retardant technology.

LBL still exhibits significant limitations in practical applications [122,124]. Firstly, this method requires multiple cycling operations to construct the film. When the target film thickness reaches dozens or even hundreds of layers, the preparation process becomes time-consuming, resulting in relatively low overall efficiency. Secondly, its application scenarios are somewhat limited. While it offers clear advantages for porous or complex-shaped materials with high specific surface areas, such as fibres and foams, its effectiveness is limited when it comes to bulk solid materials. This is because the solution finds it difficult to penetrate internally, so it can only form a surface coating. This means that it fails to achieve uniform modification throughout the entire material. Finally, scaling up the application of this method poses a challenge. The traditional, immersion-based operational model is inefficient and cannot meet the demands of large-scale, continuous industrial production.

## 4. Inorganic Clay Synergistic Bio-Based Flame-Retardant Polymer Composites

### 4.1. Sepiolite/Bio-Based Flame-Retardant System

**Figure 6:**
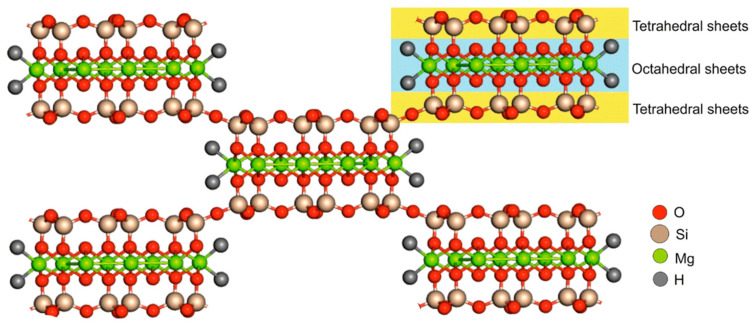
Crystal structure model of sepiolite [130] (open access).

Sepiolite (SEP) is a typical fibrous clay mineral with the chemical formula Mg_8_Si_12_O_30_ (OH)_4_ (OH_2_)_4_·8H_2_O [125,126,127]. The crystal structure model is shown in Figure 6. Its crystal structure comprises two layers of silicon–oxygen tetrahedra, with magnesium–oxygen octahedra located between them. With a 2:1 configuration arranged continuously, the theoretical surface area can reach 900 m^2^/g [128]. Sepiolite has abundant natural reserves in places like Zhangjiakou in Hebei Province and Liuyang and Xiangtan in Hunan Province in China, as well as in Turkey and Spain. Thanks to its large specific surface area, high thermal stability and porosity, it is well-suited to applications such as flame-retardant synergist, adsorbent and catalyst carrier [129].

**Figure 7:**
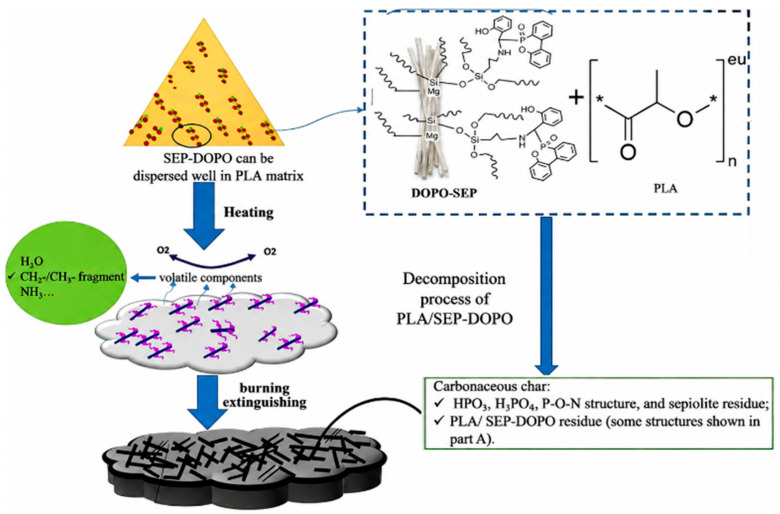
Flame retardancy mechanism of PLA/SEP-DOPO composite materials [31] (redesigned by authors).

In order to permanently strengthen the flame-retardant properties of cotton fabrics permanently, Xu et al. [131] used a layer-by-layer spraying method to coat the fabric surface with a multi-layered hybrid film made from environmentally friendly phytate (PA), sepiolite, polyaspartic acid (PASP) and Fe^3+^. This formed a dense protective layer. Research has revealed that cotton fabrics coated with hybrid films have superior flame-retardant properties and pose a reduced fire risk compared to pure cotton textiles. The afterglow and smouldering times were both recorded at 1 s, with the LOI value increasing by 44.4%. The CONE test results show a 52.6% reduction in the THR and a 73.6% decrease in the pHRR. This superior flame-retardant performance is attributed to the synergistic mechanism among sepiolite, PA, PASP, and Fe^3+^. The hybrid film composed of these four components exhibited significantly better flame-retardant performance than any of the individual components alone. Specifically, PA acts as a char-forming agent, Fe^3+^ catalyses the graphitisation of the char residue, sepiolite serves as a physical barrier reinforcing the char layer, and PASP chelates Fe^3+^ to ensure uniform dispersion. This demonstrates the synergistic flame-retardant effect of bio-based materials, such as sepiolite and phytate. Natural sepiolite is recognised as a flame-retardant additive for the PLA, but it requires a loading of at least 25 wt% to achieve adequate flame retardancy. Jiang et al. [31] therefore successfully modified sepiolite by grafting 9,10-dihydro-9-oxo-10-phosphono-10-phenoxybenzene oxide (DOPO) onto its surface through the condensation of amino groups with salicylaldehyde. The grafting ratio achieved by this process was approximately 12.8%. The SEP-DOPO was incorporated into PLA as a flame retardant. Flame retardancy testing indicates that, when the SEP-DOPO content reaches 10 wt%, the LOI value of the PLA composite material rises from 20.2% to 31.5%, thereby achieving a V-0 rating in UL94 testing. The pHRR is reduced by 40.7%, demonstrating that DOPO-grafted SEP enhances the fire resistance and thermal stability of PLA composites synergistically. The flame-retardant mechanism study suggests that the pyrolysis products of DOPO can release PO radicals in the gas phase, as inferred from TGA-FTIR measurements. Solid-state NMR analysis further reveals that SEP-DOPO generates HPO_3_/H_3_PO_3_ and forms Si–O–C bonds in the condensed phase, both of which promote char formation; a schematic diagram illustrating the flame-retardant mechanism for the PLA composite is given in Figure 7. In comparison with the physical mixture (10 wt% DOPO/SEP), the grafted system (10 wt% SEP-DOPO) exhibits a much better flame-retardant effect. Nevertheless, it remains unclear whether the performance improvement brought about by grafting should be attributed to the effectiveness of the chemical modification itself or to a genuine chemical synergy between the clay and DOPO. Because the flame-retardant performance of SEP and DOPO alone was not tested, it is difficult to quantify the individual contribution of each component to PLA. However, TGA-FTIR confirms that, in the physical mixture, DOPO decomposes prematurely, thereby largely compromising its flame-retardant action; in other words, DOPO used alone does not significantly improve the flame retardancy of PLA. This mechanistic understanding provides a reasonable explanation for the synergistic effectiveness observed in the grafted SEP-DOPO system.

### 4.2. Montmorillonite/Bio-Based Flame-Retardant System

**Figure 8:**
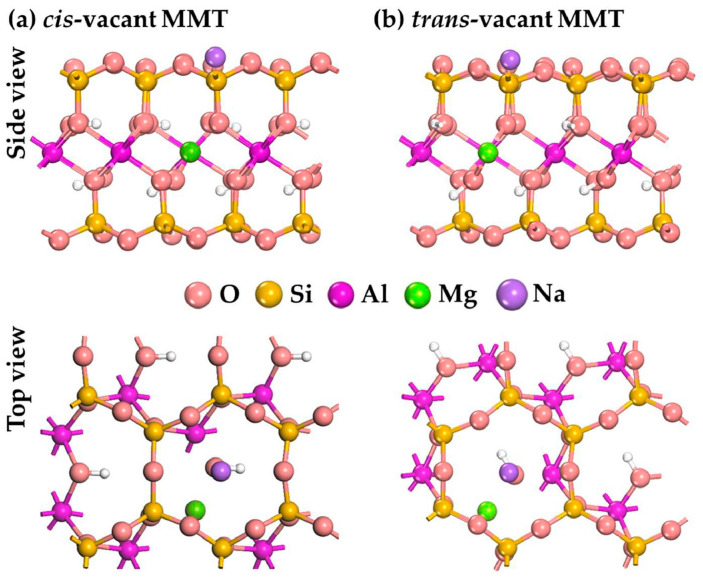
Schematic diagram of non-hydrated crystal structure model of montmorillonite [137] (open access).

Montmorillonite is a negatively charged, layered, silicate clay with the chemical formula (Na, Ca)_0.33_(Al, Mg)_2_(Si_4_O_10_)(OH)_2_·nH_2_O [132]. Figure 8 shows the non-hydrated crystal structure model of montmorillonite. Its crystal structure comprises silicon–oxygen tetrahedra and aluminium–oxygen octahedra, exhibiting a one-dimensional layered nanostructure and cation exchange properties. These characteristics confer greater modification potential upon montmorillonite, enabling it to transition from hydrophilic to more lipophilic through increased interlayer spacing and enhanced surface functional groups. This facilitates dispersion within polymer matrices [133,134]. Montmorillonite has a high specific surface area, is thermally stable and has a high surface activity. When applied as a flame retardant in polymer matrices, it helps to isolate oxygen and heat, promote char formation and enhance the material’s flame retardancy [135,136].

Yue et al. [32] introduced lignin colloidal particles into the interlayer spaces of micaceous montmorillonite. These particles significantly exfoliated and expanded the interlayer spacing of the montmorillonite. The researchers then prepared lignin/montmorillonite (LM) nanocomposites, which were used as synergists. These were then combined with intumescent flame retardants (IFRs, ammonium polyphosphate to melamine mass ratio = 5:1) to produce flame-retardant PBS composites. The text states that, when catalysed by the APP, montmorillonite and lignin form a carbonaceous silicate layer, thereby increasing residual carbon content after combustion. Furthermore, the CONE analysis shows that, compared to pure PBS, the pHRR, THR and TSP of 75P/22I/3LM (with mass fractions of PBS, IFR and LM at 75%, 22% and 3%, respectively) decreased by 57.4%, 28.7% and 33.8%, respectively. Compared with the composite containing only 3 wt% lignin, the 75P/22I/3LM composite (with 3 wt% LM) exhibits reductions of 48.4%, 19.0% and 27.8% in the pHRR, THR and TSP, respectively. Its LOI value reaches 36.5%, which is 9.28% and 16.99% higher than those of the 75P/22I/3M system (containing 3 wt% MMT alone) and the 75P/22I/3L system (containing 3 wt% lignin alone), respectively. Meanwhile, its char residue is 11.81 wt%, higher than the 9.56 wt% of the composite containing only 3 wt% lignin. These combustion data confirm a clear synergistic flame-retardant effect between lignin and MMT. During combustion, it was inferred from TGA-FTIR analysis that lignin increases the production of inert gases such as CO_2_ and H_2_, and also reacts with the phosphates derived from lignin and APP, thereby accelerating the thermal decomposition of PBS to form an initial char residue. However, no direct detection of phosphorus-containing radicals (e.g., PO·, PO_2_·) that might be generated from the bio-based system was achieved. Due to the reduction in the pore volume and surface free energy of the montmorillonite, it migrates to the surface of the expanded carbon layer. At the same time, the Al^3+^ and Mg^2+^ ions within the montmorillonite catalyse the esterification reaction between phosphates and PBS/lignin. Finally, the thermal decomposition reaction between MMT and lignin resulted in the formation of multiple layers of carbonaceous silicate on the polymer surface, which isolated the transfer and exchange of heat and combustible gases between the external environment and the substrate. Wang et al. [138] prepared cellulose/MMT bio-based plastics by subjecting cellulose hydrogels—fabricated from mixtures of cellulose solution and montmorillonite suspension—to simple press-heating treatment. Based on the favourable affinity between clay and cellulose [139], the flame retardancy of the bio-based plastic increased progressively with rising montmorillonite content. When the loading reached 20 wt%, the LOI of the bio-based cellulose/MMT plastic rose to 29.3%. The hydrogen bonding interactions between the montmorillonite clay mineral and the cellulose matrix also enhance the tensile strength of this bio-based plastic. This demonstrates the potential application value of bio-based plastic composite materials containing clay minerals in areas such as flame retardancy and packaging.

Choi et al. [140] designed an eco-friendly, high-performance, flame-retardant coating agent using cationic starch (CST) and montmorillonite nanoclay via spray-assisted, LBL assembly. This coating was then applied to polyurethane foam. The CONE testing confirmed that the HRR and THR of the coated foam were reduced by approximately half compared to the original material. Furthermore, the flame-retardant coating retained its flame-retardant properties after undergoing over 1000 compression cycles. All the results of the combustion performance tests indicate that a layer of char formed on the coated samples during the combustion process. This preserved the internal structure of the substrate and inhibited the progression of combustion. Therefore, applying LBL coating technology to polyurethane foam effectively protects it from combustion, which confirms that CST-synergistic montmorillonite exhibits excellent flame-retardant durability. Furthermore, Carosio [34] drew inspiration from this technique when preparing chitosan–MMT composites. This involved directly mixing solutions/suspensions containing oppositely charged particles and subsequently depositing them onto acrylic fibre fabrics to form multilayer coatings. This coating prevents acrylic fabrics from melting and dripping. When added at concentrations of 10% or 20%, the fabric extinguishes within seconds of ignition. According to the CONE, the coating increased the fabric’s ignition time by 46%, while shutting the pHRR and TSR by 62% and 49%, accordingly. It can be seen that the coating effectively prevents heat from transferring before and after ignition, which accelerates the formation of char on the substrate. This shows that, in coating applications, the MMT and chitosan can collaborate to deliver a condensed-phase flame-retardant effect, thus enhancing fire safety performance. In addition to clay-based flame-retardant coatings, conventional non-clay intumescent coatings also exhibit excellent flame retardancy for polymer substrates. Several studies have reported a series of highly efficient intumescent coatings constructed without clay components, which rely on classic intumescent formulations to form protective char layers during combustion [141,142]. Compared with their non-clay counterparts, clay-based intumescent coatings can further improve the compactness and thermal stability of the char layer due to the physical barrier effect of clay minerals, thereby achieving better overall flame-retardant performance.

**Figure 9:**
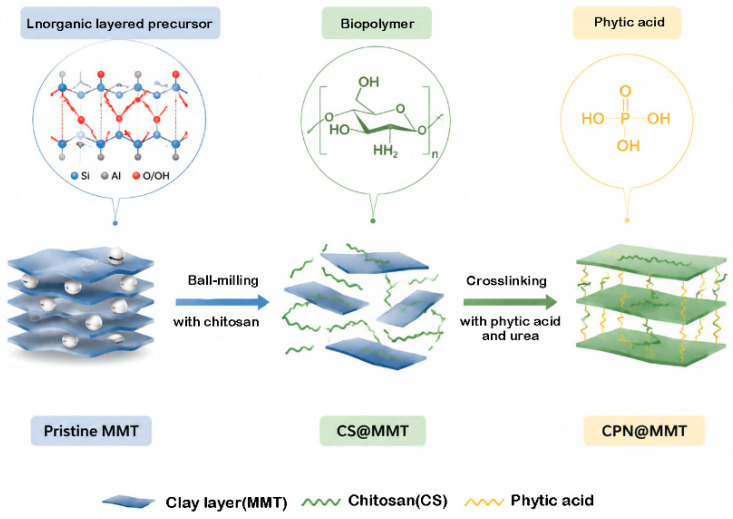
Bio-based CPN@MMT flame-retardant PU process [33] (redesigned by authors).

Han et al. [33] used ball-milling to exfoliate montmorillonite with chitosan and then co-assembled it with phytate and urea to create a bio-based supramolecular compound nanohybrid material (CPN@MMT). This material was applied to boost the flame retardance of PUA coatings. Research has demonstrated its outstanding compatibility and dispersibility within the matrix. When 3% CPN@MMT is incorporated, the pH-dependent rate of decomposition decreases by 34.1%, total suspended solids are reduced by 44.6%, and the residual carbon content increases from 7% to 22%. This is primarily due to the catalytic carbonisation and physical barrier effects of CPN@MMT, which result in a denser, more integrated carbon layer forming. At the same time, the PUA/CPN@MMT composite demonstrates exceptional impact and corrosion resistance. This further confirms the dual flame-retardant mechanism achieved through the synergistic phosphorus–nitrogen effect of montmorillonite and CPN. Figure 9 illustrates the combustion flame-retardant process.

### 4.3. Kaolinite/Bio-Based Flame-Retardant System

**Figure 10:**
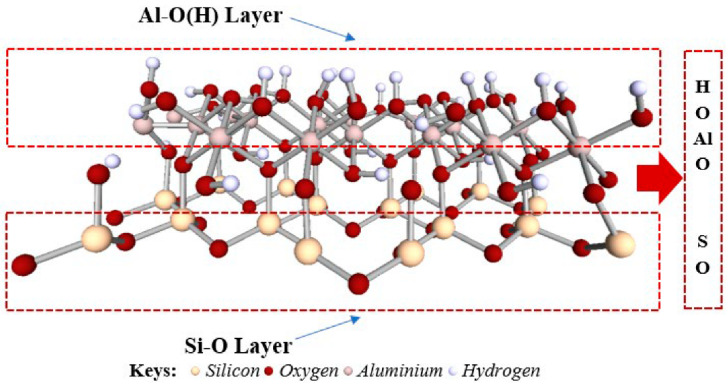
A structural model of the inner and outer layers of kaolin [146] (open access).

Kaolinite is a clay mineral composed of aluminium oxide and silicon dioxide. It is formed by the alternating arrangement of silico-oxygen tetrahedra and hydroxyaluminite octahedra in a 1:1 ratio. Figure 10 illustrates the structure of kaolin. Its theoretical chemical formula is Si_2_Al_2_O_5_(OH)_4_ and it usually occurs as compact, cryptocrystalline masses or earthy aggregates. Kaolinite is mainly found in the Chinese provinces of Jiangxi, Hebei and Hunan, as well as in Cornwall in the UK and in Georgia in the US. Natural deposits are abundant and easily accessible, as well as being low-cost. Their fine particle size, excellent dispersion and high chemical and thermal stability also make them suitable for use as refractory materials, plastic fillers and raw materials in the manufacture of ceramics and paper [143,144,145].

Sun et al. [35] successfully prepared a multidimensionally modified form of kaolinite (Mul-K_0_) using a simple supramolecular self-assembly technique to grow melamine–phytate nanosheets between the layers and on the surface of the kaolinite. This design improves upon the poor dispersion of conventional fillers by combining interlayer modification and surface grafting. SEM analysis reveals an expansion of the interlayer spacing to 3.2 nm. The Mul-K_0_ was incorporated into PUA alongside IFR (composed of a charring–foaming agent and micro-encapsulated ammonium polyphosphate). The flame retardancy and smoke suppression properties of the resulting composite material were then evaluated using oxygen–acetylene ablation testing, the LOI assessment, UL-94 vertical burning tests and CONE analysis. Research has revealed that incorporating 1 wt% Mul-K_0_ into the PUA matrix when PI-1.0Mul-K_0_ is added increases the LOI to 32.2%, while reducing the pHRR, THR and TSP by 74.6%, 46.1% and 57%, accordingly. Concurrently, tensile strength and elongation at break grew by 18% and 32%, accordingly. This flame-retardant mechanism is owed, firstly, to the material’s inherent layered crystalline structure, which serves as a physical barrier to retard heat transfer. Secondly, the P-N elements within the bio-based sheet layers catalyse with the acidic sites of the metakaolin in a synergistic way to form a graphitised carbon layer. This layer prevents the diffusion of combustible gases.

Tang et al. [36] synthesised a novel, bio-based flame retardant (V-Cc-PP), using vanillin and PA. This was used to replace conventional intumescent flame retardants (IFR, composed of ammonium polyphosphate and pentaerythritol with a mass ratio of 3:1) and enhance the flame retardancy of EP. This flame retardant was synthesised via a Schiff base reaction, after which it was modified by phosphorylation. This significantly enhanced its flame retardancy properties when combined with EP. Research has revealed that adding 4.0 wt% V-Cc-PP significantly improves the fire resistance of EP composites, reducing the pHRR by 34.2% compared to pure EP. The V-Cc-PP formulation gives EP self-extinguishing properties during combustion, whereas EP composites containing APP or IFR form melt droplets when burning. This confirms that V-Cc-PP is a more effective flame retardant than APP and IFR. Furthermore, incorporating a composite material containing 0.8 wt% kaolinite and 3.2 wt% V-Cc-PP resulted in a further decrease in the pHRR value to 38.6%. Compared to other EP composites, the EP/3.2 wt% V-Cc-PP/0.8 wt% Kaol composite material exhibits exceptional thermal stability. On the basis of results obtained from TGA-FTIR, Raman spectroscopy, and additional measurements, a reasonable inference concerning the flame-retardant mechanism was made. This is due to the layered structure of kaolinite, the carbonisation effect resulting from V-C-P decomposition producing P-N radicals, and the combined effect of chemical cross-linking between the Si-OH groups on the kaolinite surface and the P-O bonds in the bio-based flame retardant. Collectively, these factors endow the EP composite material with enhanced environmental sustainability while maintaining its high performance.

**Figure 11:**
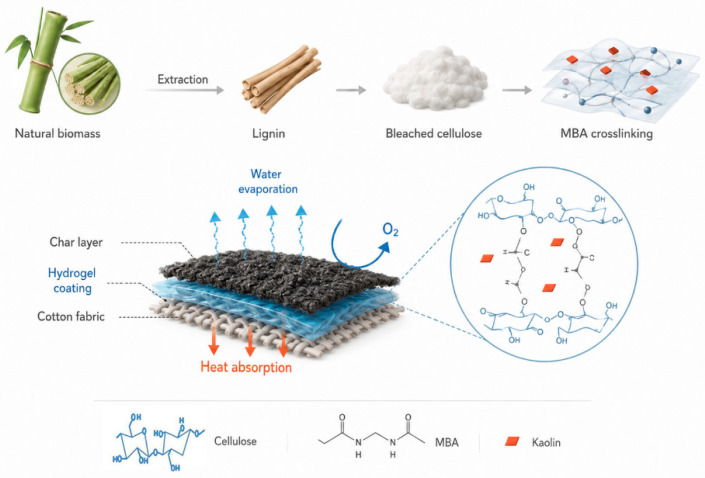
Preparation of cellulose-embedded kaolinite-MBA composite flame-retardant hydrogels [147] (redesigned by authors).

Ingtipi et al. [147] prepared kaolinite-embedded cellulose hydrogels that can soak up and hold onto a lot of moisture. Figure 11 illustrates the flame-retardant process. Research conducted using vertical flammability (VFT) and open flame (OFT) tests revealed that incorporating kaolinite improves the flame retardance of hydrogels, achieving heat dissipation rates as low as 26.6 kJ/m^2^. When chemically cross-linked with methylene bisacrylamide (MBA) as a green flame-retardant coating, hydrogels containing 2 wt% kaolinite enhance the LOI of coated cotton fabrics from 20% to 34.37%. This treatment increases the ignition delay time of cotton cloth to 19.67 s while significantly reducing the HRR and THR. Furthermore, TGA testing shows that fully thermally decomposed hydrogels can contain up to 63% residual carbon. Consequently, the interaction between the hydrogel’s water content, char formation capacity and kaolinite is synergistic, effectively retarding flame propagation in cotton fabrics. This provides a foundation for relevant research into the utilisation of kaolinite-enhanced bio-based systems in polymer flame retardancy.

### 4.4. Attapulgite/Bio-Based Flame-Retardant System

**Figure 12:**
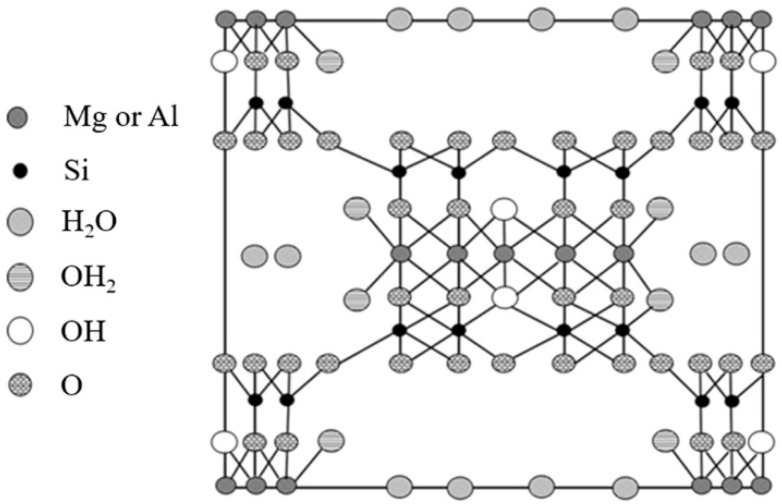
The ATP model and its plan view [152] (open access).

Attapulgite (ATP), also known as palygorskite, is a hydrated magnesium aluminium silicate clay with the theoretical molecular formula Mg_5_(Si_4_O_10_)_2_(OH)_2_(H_2_O)_4_·4H_2_O. Figure 12 shows a model of the structure of ATP. It has a rod-shaped or fibrous crystal structure and a 2:1 transitional layer chain structure [148,149]. It boasts a large specific surface area, excellent thermal stability and impressive mechanical properties. At elevated temperatures, it transforms into an insulating metal oxide layer, making it ideal for improving the flame retardancy and mechanical properties of polymer composites. Unlike other clays, attapulgite has predominantly nanoscale internal pores, giving it superior adsorption properties to other materials [150,151].

Wang et al. [153] proposed an effective, simple and environmentally friendly method of producing flame-retardant, superhydrophobic polymer-based materials. This method successfully overcomes the challenge of achieving both flame retardancy and oil/water separation. A composite coating that mimics the structure of the pearl layer was successfully constructed and applied to polyamide fibre textiles (PFTs) through the electrostatic self-assembly of chitosan (CS), PA and attapulgite. This coating forms covalent bonds between the phosphate groups of PA and the amino groups of CS, as verified by FTIR, which reveals a characteristic peak at 1030 cm^−1^ for the C-O-P bond. Meanwhile, the silanol groups (Si-OH) of attapulgite enhance interfacial bonding with PA’s phosphate groups via hydrogen bonds, as revealed by XPS, which shows a Si-O-P bond binding energy of 103.2 eV. Research has found that the coating exhibits good superoleophobicity underwater, with an underwater contact angle of 158.6°. When applied to PFT, the coating exhibits excellent flame retardancy, high thermal stability and mechanical properties. Even after 16 cycles of use and 24 h immersion in either an 80 °C, pH = 1 HCl solution or a pH = 13 NaOH solution, the coated PFT can separate corrosive oil–water mixtures exceeding 80 °C with a separation efficiency of over 96.7%. The flame-retardant mechanism of this bio-based system, which works in synergy with other inorganic clay minerals, is similar to that of flame-retardant polymers. This is possible thanks to the PA@ATP coating, which achieves biphasic synergistic flame retardancy through condensed-phase and gas-phase radical scavenging mechanisms. This method holds potential advantages in industrial wastewater treatment applications. In such reports, Kong et al. [154] prepared a novel lanthanum-modified chitosan–attapulgite composite material (La-CTS-ATP), with the optimal preparation conditions being determined through orthogonal design experiments. The adsorption performance of phosphate in aqueous solutions was investigated using adsorption kinetics, isotherms and thermodynamics. The findings revealed that the La-CTS-ATP composite material containing 8 wt% lanthanum (with a CTS to ATP ratio of 1:2) exhibited outstanding adsorption properties, achieving a monolayer adsorption capacity of 102.9 mg/g—significantly surpassing that of other lanthanum-based adsorbents. Even after five reuse cycles, the phosphate removal and desorption efficiencies remained at 72.93% and 77.44%, respectively. Therefore, La-CTS-ATP is considered a highly efficient material for removing phosphate, which is of significant importance for environmental protection.

**Figure 13:**
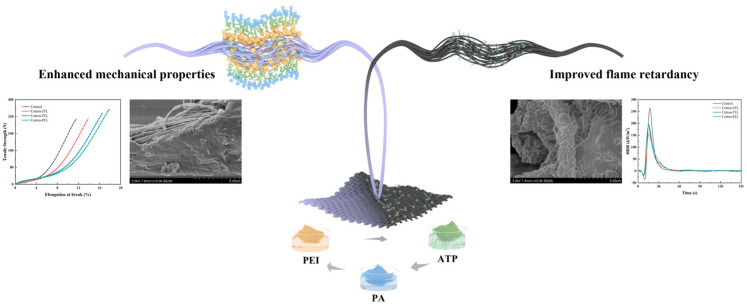
A synergistic eco-friendly, clay-based flame-retardant coating is applied to flame-retardant cotton fabrics [37] (open access).

Kang et al. [37] used a layer-by-layer assembly technique to create a flame-retardant coating, with polyethyleneimine (PEI), the ATP and PA serving as raw materials. This coating was found to effectively enhance the thermal stability of cotton fabrics, increasing the LOI by 27% and reducing the pHRR by up to 41%. At the same time, the deposition of the coating significantly increased the char residue content. Furthermore, the tensile strength of flame-retardant cotton fabrics has increased by 20% along the length and 32% across the width. The flame-retardant mechanism involves the synergistic action of the ATP and PA. The chemical composition of the char residue was analysed by FTIR, which revealed the presence of C–O–C, P–O, and Al–O–Si bonds. Raman spectroscopy was employed to determine the ID/IG ratio in order to evaluate the degree of graphitisation of the char. During this process, the PA decomposes into polyphosphoric acid and phosphoric acid at high temperatures, acting as an acid source. These phosphoric compounds then undergo dehydration, which promotes the carbonisation of cellulose. The SiO_2_, MgO and Al_2_O_3_ formed by the thermal decomposition of ATP act together as a physical barrier, enclosing the gas and preventing heat transfer. This results in the carbon layer expanding with bubbles and a significant increase in the degree of graphitisation of the residual carbon. It was also observed by the TGA-FTIR that the use of foaming agents in coated cotton fabrics effectively diluted the O_2_ concentration through the generation of NH_3_ gas. This is clearly visible in Figure 13. This novel eco-friendly coating simultaneously enhances the flame retardancy and mechanical properties of cotton fabrics, offering a fresh approach to flame-retardant treatment for cotton textiles.

### 4.5. Layered Double Hydroxide/Bio-Based Flame-Retardant System

**Figure 14:**
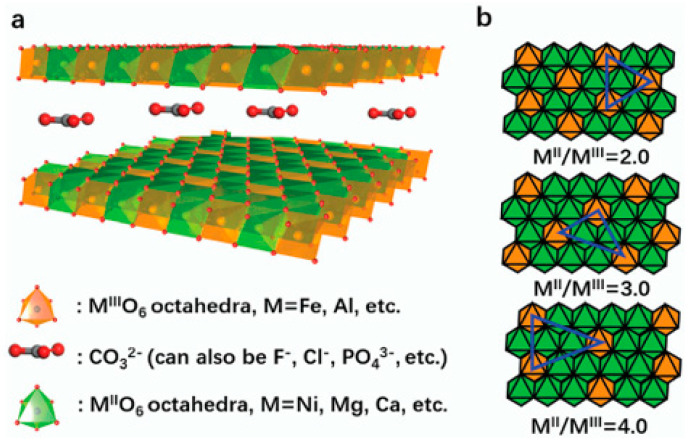
(**a**) Layered structure of LDHs. (**b**) Evolution of metal cation distribution in LDH nanosheets with increasing M^2+^/M^3+^ ratio from 2.0 to 4.0, showing the single-atom dispersion characteristics of M^3+^ sites [158] (open access).

Layered double hydroxides (LDHs) is a category of inorganic materials assembled through non-covalent bonds between a host layer and interlayer anions. Its structure resembles that of hydromagnesiumite, consisting of positively charged layers. It is also referred to as anionic clay or hydromagnesiumite-type compounds [155,156,157]. Figure 14 shows a structural model of LDHs.

In clay/bio-based synergistic systems, LDHs is not inert, but a multifunctional and designable active nanoscale platform. This is demonstrated by its ability to exchange interlayer anions with various functional anions. Bio-based anions (e.g., PA) can be loaded into its interlayers through ion exchange [159,160]. This method tackles the problems associated with bio-based molecules, such as their tendency to migrate and their poor thermal stability. The layered structure of LDHs itself serves as an excellent physical barrier. The metal ions within the layers (e.g., Al^3+^) and the intercalated phosphorus-based biomolecules work together to catalyse the dehydration and carbonisation of the polymer matrix. At high temperatures, the metal oxides decompose and absorb particulate matter from the smoke, catalysing the conversion of CO into CO_2_ and significantly reducing both smoke volume and toxicity [38,161,162].

**Figure 15:**
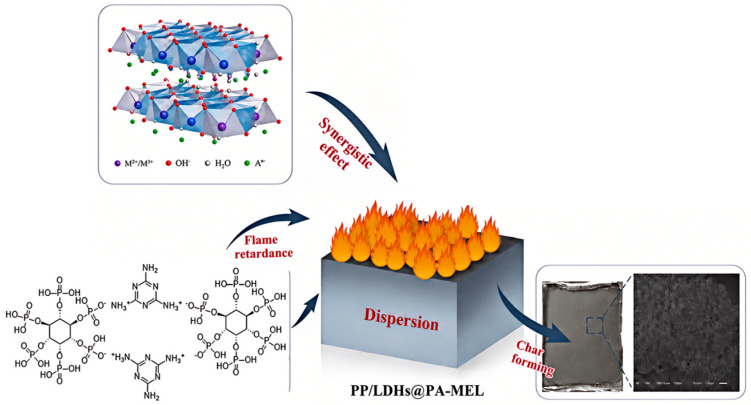
Schematic of LDHs@PA-MEL for highly efficient flame retardation of PP [38] (redesigned by authors).

The one-pot method is an efficient approach for synthesising LDHs composite flame-retardants under certain conditions. As shown in Figure 15, Yan et al. [38] used PA, melamine (MEL) and layered double hydroxides (LDHs-C) as flame-retardant precursors to synthesise intercalated/surface-modified flame retardants (LDHs@PA-MEL) using a one-pot method. In this method, the PA was intercalated into the layers in an anionic form. This flame retardant demonstrates excellent dispersibility in the PP thanks to the dual modification provided by the PA and MEL. After adding 20 wt%, it was found that the LOI of the PP/LDHs@PA-MEL composite material increased to 29.6%, higher than the 24.8% LOI of the PP/LDHs-C composite material. Compared to pure PP, the pHRR, THR and TSP declined by 63.5%, 26.2% and 43.7%, respectively. This is owing to the addition of PA, which endows the flame-retardant material with high-quality charring capabilities. Modifying the material with PA and MEL has effectively resolved the issues of uneven bonding and weak charring ability in PP/LDHs.

In the area of flame-retardant PP, significant work has also been undertaken by Wu et al. [39]. They used a co-precipitation method to synthesise an environmentally friendly, novel flame retardant based on SLS-modified layered double hydroxide (LDH-LS). They then prepared PP/LDH-LS composites via melt blending. The successful modification of SLS was characterised using the FTIR, XRD and XPS techniques and was achieved by adsorption onto the LDH surface. Furthermore, the enhanced hydrophobicity of the LDH-SLS composite indicates its excellent compatibility with the PP matrix. Research into flame retardancy testing indicated that the pHRR, THR and TSP values of PP composite materials decreased by 62.9%, 25.1% and 43.3%, accordingly. Following the incorporation of 20 wt% LDH-LS, the PP/LDH-LS composite showed an LOI of 29.4% and obtained a UL-94 V-0 classification. By contrast, neither the PP/LDH-20 nor the PP/SLS-20 composites achieved a rating in the UL-94 test. Compared with neat PP, the PP/SLS-20 composite containing 20 wt% SLS alone showed reductions in the pHRR, THR, and TSP of 37.65%, 14.25%, and 6.7%, respectively; the PP/LDH-20 composite with 20 wt% LDH alone exhibited reductions of 33.25%, 25.11%, and 20.15%, respectively. These reductions are much less pronounced than those achieved by the combination of both components in the PP/LDH-LS-20 composite. This clearly demonstrates the excellent synergistic flame-retardant effects exhibited by the LDH and LS. This improvement is attributed to the synergy between LDH and LS. Their combined action promotes the formation of a coherent, thermally stable char residue, which in turn enhances the overall flame-retardant performance. These layers create a physical barrier that effectively prevents the exchange of oxygen and heat between the PP matrix and the external environment.

### 4.6. Other Inorganic Clay Minerals/Bio-Based Flame-Retardant System

Inorganic clay/bio-based systems comprising other inorganic clay minerals, such as vermiculite, talc and serpentine, are a promising area of research within the field of polymeric green flame retardants. To date, little prior research has been conducted in this area. As silicate clays, these materials all exhibit high-temperature resistance. Although they cannot be modified via ion exchange like the LDH, or undergo the rapid organication seen with the montmorillonite, binding with bio-based molecules relies more on physical adsorption or weak hydrogen bonding. Nevertheless, following physical and chemical modification, they can be evenly dispersed throughout the polymer matrix, providing a physical barrier and achieving synergistic flame retardancy.

Vermiculite is a layered silicate clay mineral that expands at high temperatures. It has the chemical formula (Mg, Ca)_0.7_(Mg, Fe^3+^, Al)_6_(Si, Al)_8_O_20_(OH)_4_·8H_2_O [163,164]. It belongs to the 2:1 class of inorganic mineral clays [165,166]. Vermiculite has a high cation exchange capacity and excellent cation exchange and adsorption properties [167,168]. Its unique properties make it ideal for use in flame-retardant polymers, and it can be blended with char-forming, bio-based flame retardants. At elevated temperatures, it forms a fluffy, porous insulating layer. This robust, expanded carbon layer blocks the transfer of external heat inward effectively, thereby delaying the thermal decomposition of the polymer matrix [169,170].

**Figure 16:**
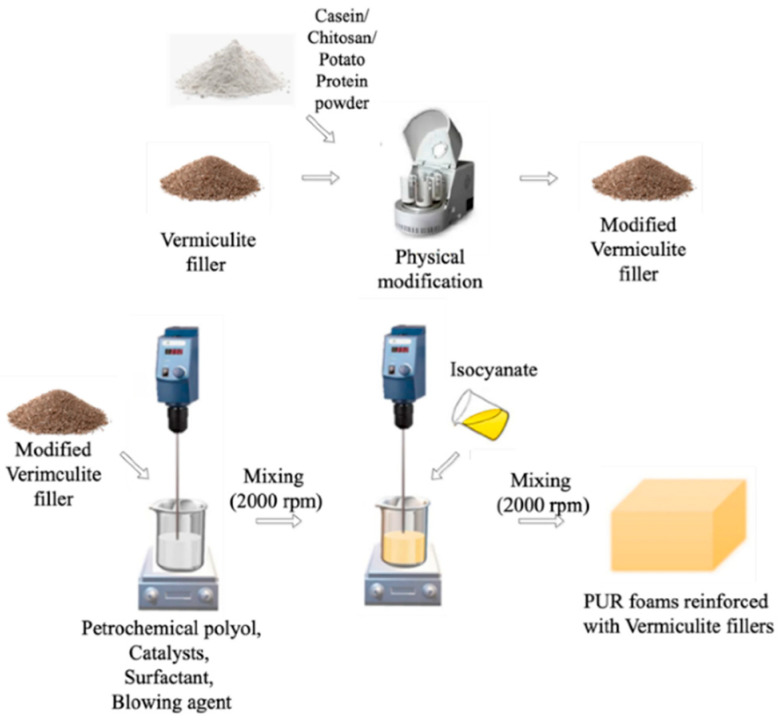
Schematic diagram of synthetic PU composite materials [171].

Miedzińska et al. [171] used casein, chitosan and potato protein to modify the surface of vermiculite, forming a flame-retardant composite filler. This composite material was then used to fill polyurethane foam (PU). The flame retardancy and mechanical properties of the composite material are then explored. The PU incorporating vermiculite coated with modified chitosan and casein protein exhibited the most pronounced improvement in mechanical and fire-retardant performance: compressive strength increased by 6–18%, flexural strength by 2–10%, and toughness by 1–5%, while the TSP and the pHRR decreased by approximately 34% and 25%, respectively. It demonstrates that modified vermiculite filler can effectively improve the flame retardance and mechanical properties of PU, providing a concrete, feasible solution for the flame-retardant modification of PU. Figure 16 illustrates the manufacturing process.

## 5. Conclusions

This paper reviews the current applications of inorganic clay minerals and bio-based flame-retardant polymers for which research has been published. It presents a systematic and comprehensive overview of the knowledge base, covering everything from the fundamental mechanisms and preparation methods to the specific applications of polymer materials. The majority of fundamental research has focused on multi-scale structural design and flame-retardant composite preparation methods. A novel, highly effective and eco-friendly flame-retardant composite material has been developed. This is by integrating the physical barrier properties and catalytic carbonisation effects of natural inorganic mineral clays. It also incorporates the eco-friendly nature and vapour–condensate interactions of bio-based flame retardants. The method of preparation directly affects the interfacial bonding and micro-dispersion structure of the composite material, which is key to regulating its flame-retardant properties. Therefore, a balance must be struck between efficiency and structural controllability on the one side, and the complexity of component design formulations on the other, taking into account requirements such as modified dispersion properties and surface coating, as well as the construction of core–shell structures and the characteristics of bio-based molecules (e.g., thermal stability and molecular size). This remains an important direction for future research.

Regarding synergistic flame-retardant mechanisms, there is the reinforcement of physical barriers, the optimisation of catalytic char formation pathways and the effects of radical trapping and dilution. During the high-temperature decomposition of clays such as montmorillonite and sepiolite, a dense, stable carbon layer of silicate forms. At the same time, bio-based components such as phytate and chitosan decompose to produce inert gases (e.g., NH_3_ and H_2_O), which dilute oxygen and combustible gases while simultaneously trapping free radicals. Therefore, the synergistic effect of inorganic clay minerals and bio-based systems is more than just the sum of their parts. It is the result of the interplay between the components of the inorganic bio-based system. When bio-based flame retardants are ineffective or of poor quality in catalysing polymer char formation, inorganic clay minerals such as LDH and modified sepiolite can serve as highly effective catalysts. During combustion, these minerals promote the cross-linking and aromatisation of bio-based molecules, resulting in the generation of a high-quality, graphitised, expended char layer.

Although the combination of inorganic clay minerals and bio-based flame-retardants offers significant advantages over traditional halogenated flame retardants, there are still many issues to address in industrial applications, particularly with regard to poor interface compatibility and dispersibility, before it can meet high-performance requirements. Although binary or multi-component flame-retardant systems are highly efficient, most inorganic clays have hydrophilic surfaces and poor compatibility with non-polar polymer matrices, which makes them prone to aggregation within the matrix. Introducing bio-based molecules may further exacerbate the difficulty of interfacial bonding and the complexity of flame-retardant formulation design. Techniques such as LBL deposition and co-precipitation impose high demands on equipment and design. Determining the optimal flame-retardant formulation ratio is challenging, with most designs relying on orthogonal experiments. The synergistic flame-retardant mechanism is not fully understood. Current research methods for studying flame retardants, such as thermogravimetric–infrared coupling and pyrolysis-gas chromatography–mass spectrometry coupling, cannot accurately reflect the chemical transformation process that occurs during combustion. These methods can only provide information on the functional groups of gaseous product molecules, failing to yield precise chemical structures.

Therefore, future research should prioritise the development of smarter, higher-efficiency, more sustainable approaches. One approach could be to establish covalent bonds or strong hydrogen bond networks between clay, bio-based molecules and polymer matrices to achieve the integration of flame retardancy and impact resistance, using molecular dynamics simulations, machine learning and other methods to screen and predict the optimal clay bio-based molecular designs and their ideal blending ratios with polymers. Additionally, a real-time online analytical system for characterising polymer combustion processes should be developed. This system should enable the real-time analysis of flame-retardant performance (e.g., thermal and smoke release) and the actual combustion behaviour of polymer composites, while simultaneously determining the chemical structures of substances in both condensed and gaseous phases. This will establish novel methods for the online analysis of actual combustion processes.

## Figures and Tables

**Table 1:** Summary of various clay-based, bio-derived flame-retardant polymer systems.

Clay/Bio-Based Flame Retardant	Polymer Matrix	LOI (%)	UL-94	pHRR Reduction (%)	THR Reduction (%)	TSP (m^3^)	Ref.
10DOPO-SEP	PLA	31.5	V-0	40.7	43.1	/	[31]
22I/3LM	PBS	36.5	V-0	57.4	28.7	33.8	[32]
3CPN@MMT	PUA	/	/	34.1	19.6	44.6	[33]
20CH/MMT	Acrylic fibre	29.3	/	62.0	49.0	49.0	[34]
PI-1.0MK_0_	PUA	32.2	/	74.6	46.1	57.0	[35]
3.2V-Cc-PP/0.8Kaol	EP	27.8	/	38.6	/	/	[36]
PEI/ATP/PA	cotton fabric	27.0	/	41.0	22.6	/	[37]
20LDHs@PA-MEL	PP	29.6	V-0	63.6	26.2	43.7	[38]
LDH-LS	PP	29.4	V-0	62.9	25.1	43.3	[39]

**Table 2:** Synthesis schemes of various inorganic clay/bio-based flame-retardant systems.

Clay/Bio-Based Flame Retardant	The Synthetic Route of Flame Retardants	The Preparation Scheme of Flame Retardants	Ref.
DOPO-SEP	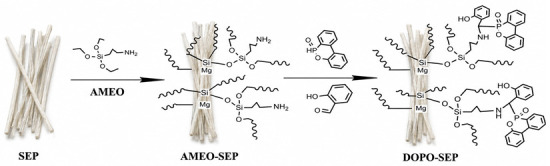	SEP-DOPO was synthesised by reacting SEP with excess AMEO, followed by 4-hydroxybenzaldehyde and DOPO in toluene at 90 °C under nitrogen atmosphere.	[31] Redesigned the image
I/LM	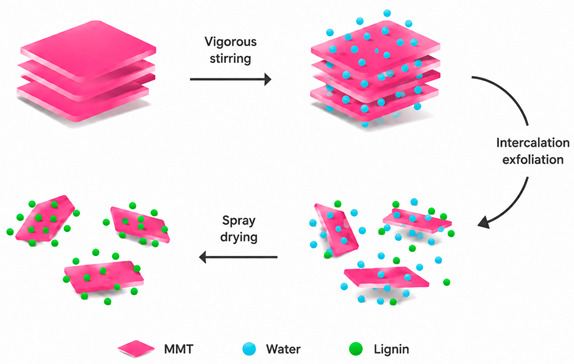	Lignin/MMT nanocomposites were prepared by mixing lignin solutions with adjusted pH (3.5–12.0) into ultrasonically dispersed MMT suspension, followed by spray drying.	[32] Redesigned the image
CPN@MMT	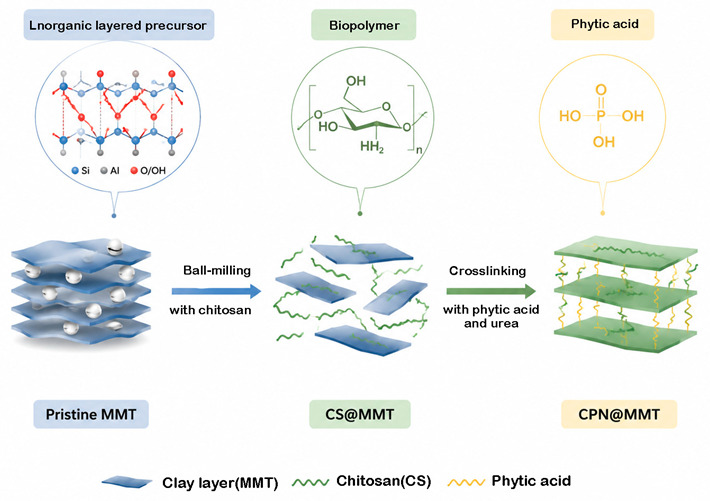	CPN@MMT nanohybrid was synthesised by ball-milling montmorillonite with chitosan, followed by co-assembly with phytic acid and urea.	[33] Redesigned the image
CH/MMT	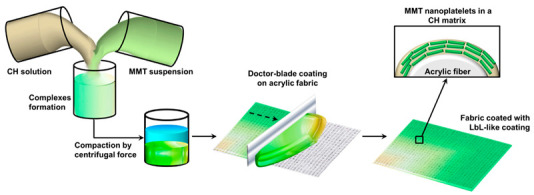	The CH/MMT gel was prepared by mixing chitosan solution and MMT suspension (1:1 *v*/*v*) under magnetic stirring for 20 min, followed by centrifugation at 4400 rpm for 5 min.	[34] open access
Mul-K_0_	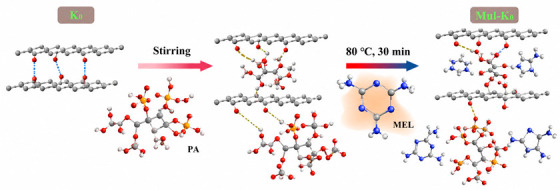	Mul-K0 was synthesised by sequentially modifying K0 with phytic acid (PA) and melamine (MEL) in an aqueous ethanol solution at 80 °C under pH 4–5.	[35] Redesigned the image
V-Cc-PP/Kaol	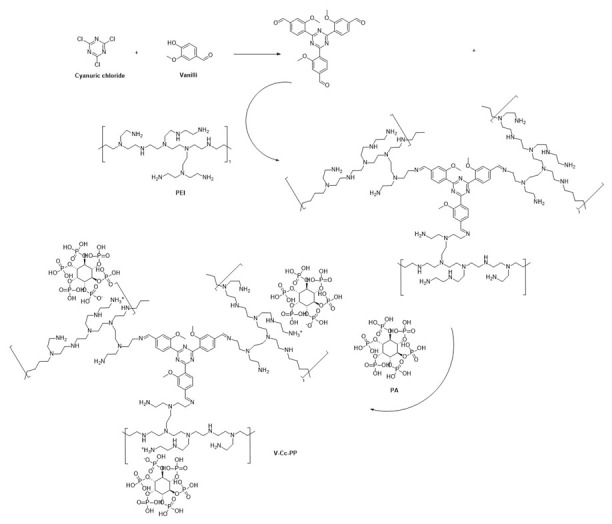	V-Cc-PP was synthesised by sequentially reacting cyanuric chloride with vanillin, followed by polyethyleneimine (PEI) and sodium phytate (PA-Na) in organic solvents under heating. Subsequently, it was compounded with kaolinite to obtain V-Cc-PP/Kaol.	[36] Redesigned the image
PEI/ATP/PA	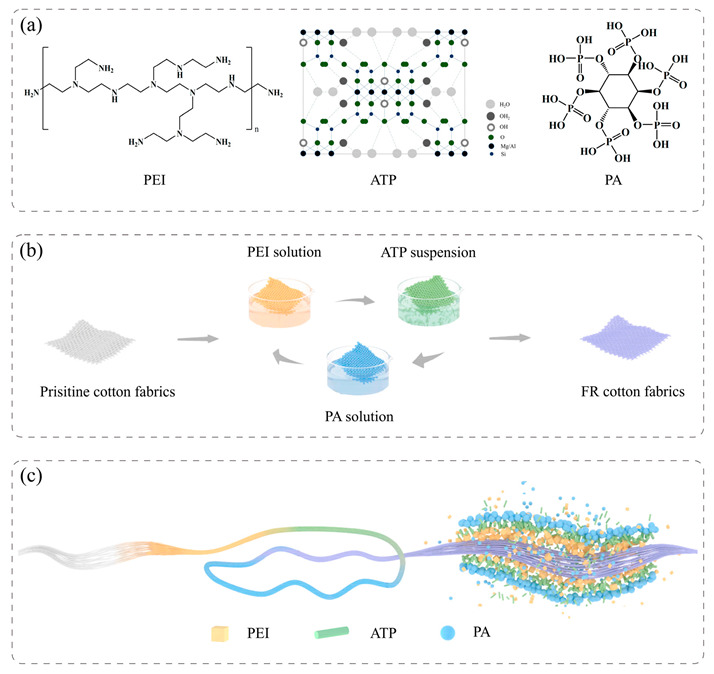	(a) Chemical structures of polyethyleneimine (PEI), hydrochloric acid-treated attapulgite (ATP), and phytic acid (PA), the key components for layer-by-layer (LbL) assembly; (b) schematic diagram of the LbL assembly process of these components on pristine cotton fabrics to produce flame-retardant (FR) cotton fabrics; (c) structural and morphological changes of a single cotton fiber after the LbL treatment.	[37] open access
LDHs@PA-MEL	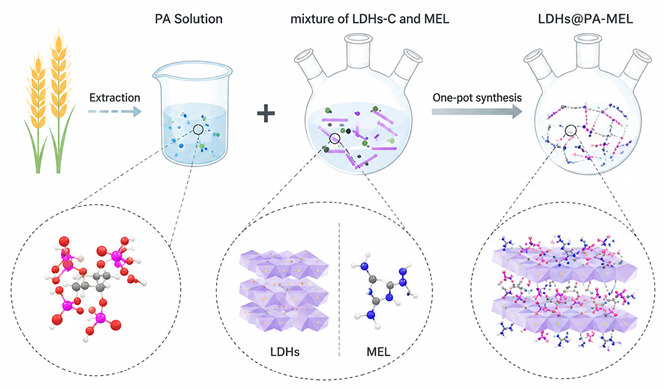	MgAl–CO_3_–LDHs (LDHs-C) with a Mg/Al molar ratio of 3.0 was prepared by a hydrothermal method. LDHs@PA-MEL was then synthesised via a one-pot reaction of LDHs-C with melamine (MEL) and phytic acid (PA) at 70 °C in the presence of NaOH.	[38] Redesigned the image
LDH-LS	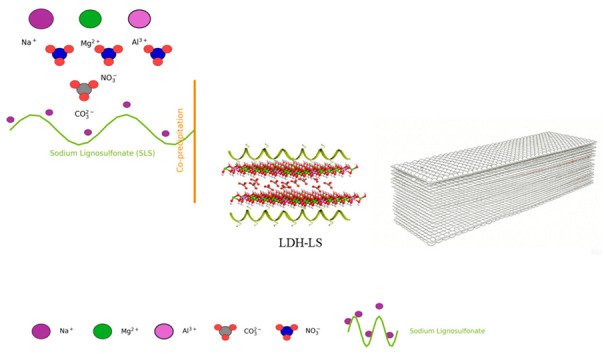	LDH-LS was synthesised by dropwise adding Mg(NO_3_)_2_·6H_2_O and Al(NO_3_)_3_·9H_2_O into a solution of sodium lignosulfonate (SLS) and Na_2_CO_3_ under pH 9–10, followed by dynamic crystallisation at 90 °C for 6 h, filtration, washing, and drying at 80 °C for 24 h.	[39] Redesigned the image

**Table 3:** Summary of primary flame-retardant mechanisms in inorganic clay minerals.

Flame-Retardant Mechanism	Mode of Flame Retardancy	Flame-Retardant Properties	Ref.
Physical barrier	A protective, densified carbon layer with a ‘carbon-silicate’ structure that exhibits a nano-dispersive effect is formed.	Block the transfer of heat and material, as well as the circulation of oxygen and other combustible gases. This will delay the combustion process.	[45,48]
Reducing the fluidity of the matrix	Increase the viscosity of the polymer melt in order to prevent the large-scale diffusion of molecular chains.	The flame suppresses the melting and dripping of the substrate, thus preventing the fire from spreading.	[46,48]
Free radical scavenging (weak)	Capturing gas-phase free radicals through impurities, such as excess iron compounds, within clay minerals.	It inhibits chain reactions in combustion and exhibits a very slight gas-phase flame-retardant effect.	[47]

**Table 4:** Several commonly used bio-based flame retardants and their primary mechanisms.

Bio-Based Materials	Principal Ingredients	The Predominant Flame-Retardant Mechanism	Typical Application Polymers	Ref.
PA	Phosphorus-rich organic acids	A strong acid source is used for highly efficient catalytic dehydration to form carbon.	PLA, EP, and fibre membrane	[55,62]
Chitosan (CS)	Aminoglycoside-containing polysaccharide	It can be used as a carbon and gas source. It possesses excellent carbonisation properties and can be used in combination with acid sources.	PLA, PU, and rubber	[63,64]
Lignin	Three-dimensional, complex phenolic polymers	It can be used as a carbon source. Due to its rich aromatic ring structures, it can produce a high residual carbon content.	PLA, PP, and PBS	[65,66]
Starch and its derivatives	Polyhydroxy polysaccharide	Carriers of carbon and acid sources.	PLA, PP	[67,68]
DNA	Deoxyribonucleic acid (DNA) contains phosphorus and nitrogen	Natural IFR system. The acid source is phosphate, the carbon source is deoxyribose, and the nitrogen source is the nitrogenous bases.	Cotton fabrics, PLA film	[69,70]
Tannic acid (TA)	Polyphenolic compounds	A carbon source and a metal ion chelating agent. It easily cross-links with metal ions to form a stable network structure that promotes carbon formation.	Polymer foam, coating	[71,72,73]

## Data Availability

No new data were created or analysed in this study.

## Abbreviations

Abbreviation	Full name
PLA	Polylactic acid
PBS	Polybutylene succinate
PUA	Polyurethane acrylate
PU	Polyurethane
PP	Polypropylene
EP	Epoxy resin
AMEO	3-Aminopropyltriethoxysilane
PVC	Polyvinyl chloride
TA	Tannic acid
APP	Ammonium polyphosphate
MA	Melamine
IFR	Intumescent flame retardant
SEP	Sepiolite
DOPO	9,10-Dihydro-9-oxa-10-phosphaphenanthrene-10-oxide
DDM	4,4′-diaminodiphenylmethane
MMT	Montmorillonite
CF	Chicken feather protein
PASP	Polyaspartic acid
PA	Phytic acid
MEL	Melamine
PEI	Polyethyleneimine
ATP	Attapulgite
LDH	Layered double hydroxide
LBL	Layer-by-layer self-assembly
SLS	Sodium lignosulfonate
LS	lignin sulfonate
LOI	Limiting oxygen index
HRR	Heat release rate
pHRR	Peak heat release rate
THR	Total heat release
TSP	Total smoke production
UL-94	Underwriters Laboratories 94 vertical burning tes
MH	Magnesium hydroxide
ATH	Aluminium hydroxide
CS	Chitosan
CST	Cationic starch
CONE	Cone calorimeter
TGA	Thermogravimetric analysis
SEM	Scanning electron microscopy
XPS	X-ray photoelectron spectroscopy
FTIR	Fourier transform infrared spectroscopy
XRD	X-ray diffraction
VFT	Vertical flame Test
OFT	Open flame Test
MBA	Methylene bisacrylamide
PFT	Polyamide fibre textiles
